# Patient socio-demographics and clinical factors associated with malaria mortality: a case control study in the northern region of Ghana

**DOI:** 10.1186/s12936-024-05038-2

**Published:** 2024-08-04

**Authors:** Nana Yaw Peprah, Wahjib Mohammed, George Asumah Adu, Dora Dadzie, Sammy Oppong, Seidu Barikisu, Joel Narh, Stephen Appiah, James Frimpong, Keziah L. Malm

**Affiliations:** 1 Public Health Division, National Malaria Elimination Programme, Ghana Health Service, Accra, Ghana; 2 Regional Health Directorate, Northern Region, Ghana Health Service, Accra, Ghana

**Keywords:** Case control, Clinical care, Ghana, Malaria mortality, Northern Region, Socio-demographics

## Abstract

**Background:**

Ghana is a malaria-endemic country with the entire population at risk. The Northern region of the country recorded the highest malaria case fatality rate (CFR) for two consecutive years: 1.11% in 2013 and 1.07% in 2014. Even though the National Malaria Elimination Programme (NMEP) has achieved a reduction in malaria mortality, the existence of high case fatality in the Northern region was alarming. This study, therefore, aimed to determine the factors associated with malaria mortality in the northern region of Ghana to institute control measures.

**Methods:**

An unmatched case control study was conducted from July 2015 to August 2015. The study population consisted of patients admitted to health facilities for severe malaria in the Northern region of Ghana. A case was defined as a patient diagnosed with severe malaria at an eligible health facility who died as a result of malaria. A control was a patient diagnosed with severe malaria admitted to an eligible health facility who did not die. Health facilities that recorded CFRs of 1.0% and above were randomly sampled for this study, after which, 10 cases and 20 controls were recruited from each health facility. Information on cases and controls was then abstracted from hospital records using an electronically deployed abstraction tool. Continuous variables were expressed as means and medians, and categorical variables as frequencies and proportions. Multivariable logistic regression was used to assess the strength of the association between malaria mortality and factors predictive of malaria mortality. A p-value of < 0.05 was considered statistically significant.

**Results:**

In all, a total of 95 cases and 190 controls participated in this study. The median ages of cases and controls were 4.1 years (IQR = 21.6) and 5.7 years (IQR = 18.2), respectively. Fifty-four (56.8%) cases were females, while 93 (49.0%) of the controls were females. Factors associated with malaria mortality included: duration of hospital stay less than 24 h [aOR: 12.0, 95% CI (5.9–24.6)], severe pallor [aOR: 2.3, 95% CI (1.1–4.6)], children under 5 years [aOR: 2.8, 95% CI (1.4–5.6)], oral Artesunate/Amodiaquine administration [aOR: 0.4, 95% CI (0.2–0.9)] and sepsis as an additional diagnosis [aOR: 4.1, 95% CI (1.8–9.5)].

**Conclusion:**

Predictors of malaria mortality in the Northern region include children under 5 years, severe pallor, sepsis as an additional diagnosis, and use of oral anti-malarial. Patients with severe pallor and sepsis as co-morbidities should receive proactive management. The NMEP and its partners should implement measures to strengthen the referral system, anaemia prevention and management, and retrain health workers on malaria case management. Malaria control interventions targeted at under five children in the region should be reviewed and enhanced.

## Background

Malaria remains a major public health burden in developing countries, despite tremendous control efforts. Globally, the Africa Region accounted for 94% of all malaria cases (233 million cases) and 95% of all malaria deaths (580 000 deaths), with 78% of all malaria deaths occurring among children under the age of five and pregnant women in 2022 [1]. Ghana is a malaria-endemic country, with the entire population at risk. Ranked among the top 15 nations globally in terms of malaria burden, it accounted for 2.2% of global malaria cases as well as 2% of global malaria-related fatalities. It is responsible for around 4% of malaria cases in the West African region [2]. Notwithstanding, Ghana has made notable progress in the malaria fight over the past decade. Malaria prevalence decreased from 27.5% in 2011 [3] to 8.6% in 2022 [4], with the Greater Accra Region recording the lowest prevalence of 2% and the Oti Region recording the highest prevalence of 10%. The number of confirmed malaria cases per 1000 population decreased from 192 in 2019 to 159 in 2022, while the number of malaria deaths also decreased from 2,799 in 2012 to 151 in 2022 [5].

Despite the progress, some parts of the country bear a disproportionally high burden of the disease, one of which is the Northern Region of Ghana. In 2022, the region recorded a prevalence of 10.6% [4]. In the same year, malaria accounted for 26.1% of OPD attendance, 32.5% of admissions, and 0.37% of all deaths. This represents a reduction in the malaria burden in the region compared to 2012, when malaria accounted for 51.4% of OPD attendance, 8.73% of admissions, and 25.2% of all deaths []. The malaria deaths in the region 10 years ago were the most concerning, with the Northern region consecutively recording the highest case fatality rates (CFR) in 2013 and 2014 (1.11 and 1.07, respectively) []. This region is one of the few that benefits from context-specific malaria control interventions in addition to those rolled out countrywide. Hence, the reasons for the comparatively higher CFR were not immediately clear.

A study by Aziz et al*.* [6] in the Northern Region of Ghana revealed referral status, age, distance, treatment, and length of stay on admission as relevant predictors of malaria mortality [7]. Other factors that have been associated with malaria mortality, include non-use of mosquito nets, respiratory diseases, seizures, hypoglycaemia, incorrect drug administration, sex, delayed diagnosis, falciparum malaria, and poor immunity [8, 9]. Given that diverse factors influenced malaria mortality, it was necessary to identify the specific factors driving malaria mortality in the Northern region to guide interventions aimed at mortality reduction in the region.

Consequently, in 2015, the National Malaria Elimination Programme (NMEP) explored the factors associated with malaria mortality in the Northern Region of Ghana to inform policy decisions on averting malaria mortalities.

## Methods

### Study area

This study was conducted in the old Northern Region of Ghana, which was later divided into three regions, namely the Northern, Savannah, and North East regions in 2019. Prior to the split, the Northern Region was the largest of Ghana's ten regions. It covered an area of 70,384 square kilometers, or 31 percent of Ghana’s entire surface area. The region, which was largely rural, was divided into 26 districts and had Tamale as its capital. The region had a low population density of about 2,479,461 [10].

It is bordered to the north by the Upper West region and the Upper East region, to the east by the eastern Ghana-Togo international border, to the south by the Black Volta River and the Volta region, to the north-west by the Upper West region and Burkina Faso, and to the west by the western Ghana-Ivory Coast international border. There is one district hospital or polyclinic in each district, along with a number of health centers and Community Health and Planning Services (CHPS) across each district. Due to its proximity to the Sahel and the Sahara, the Northern Region is much drier than the southern areas of Ghana, with temperatures varying between 14 °C (59 °F) at night and 40 °C (104 °F) during the day.

### Study design

An unmatched case–control study was conducted from July 2015 to August 2015. The study population consisted of patients diagnosed with severe malaria between January 2014 through December 2014 in health facilities in the Northern region with a malaria case fatality rate of 1% or more. Patient records were subsequently retrieved from registers and folders using an electronically deployed abstraction tool for analysis. No participant was interviewed. A case was defined as a patient diagnosed with severe malaria at an eligible health facility (health facility with CFR ≥ 1%) in the Northern region from January 2014 through December 2014 who died as a result of malaria. A control was a patient diagnosed with severe malaria admitted to an eligible health facility in the Northern region from January 2014 through December 2014 who did not die. All patients with a diagnosis of severe malaria, except those with severe co-morbid, non-communicable disease or severe co-morbid non-malarial febrile infections, were eligible for participation.

### Sample size determination

The sample size was estimated using the formula:

$$n\, = \,{{\left[ {{{Z\alpha } \mathord{\left/ {\vphantom {{Z\alpha } {2\sqrt {2pq} }}} \right. \kern-0pt} {2\sqrt {2pq} }}\, + \,Z_{\beta } \sqrt {\left( {p_{1} q_{1} + \,p_{0} q_{0} } \right)} } \right]^{2} } \mathord{\left/ {\vphantom {{\left[ {{{Z\alpha } \mathord{\left/ {\vphantom {{Z\alpha } {2\sqrt {2pq} }}} \right. \kern-0pt} {2\sqrt {2pq} }}\, + \,Z_{\beta } \sqrt {\left( {p_{1} q_{1} + \,p_{0} q_{0} } \right)} } \right]^{2} } {\left( {p_{1} - p_{0} } \right)}}} \right. \kern-0pt} {\left( {p_{1} - p_{0} } \right)}}^{2}$$

OR

$$n\, = \,{{2pq\left( {{{Z\alpha } \mathord{\left/ {\vphantom {{Z\alpha } 2}} \right. \kern-0pt} 2} + Z_{\beta } } \right)^{2} } \mathord{\left/ {\vphantom {{2pq\left( {{{Z\alpha } \mathord{\left/ {\vphantom {{Z\alpha } 2}} \right. \kern-0pt} 2} + Z_{\beta } } \right)^{2} } {\left( {p_{1} \, - \,p_{0} } \right)}}} \right. \kern-0pt} {\left( {p_{1} \, - \,p_{0} } \right)}}^{2}$$

n: sample size for one sample.

z_α/2_: z value for a two-sided test corresponding to the chosen αα.

z_β_: z value for a one-sided test for the chosen β.

p_1_: proportion of cases exposed.

p_0_: proportion of controls exposed.

p*: mean estimated proportion (p_1+_p_0_)/2.

q = 1-p.

Where Z = 1.96 is the standard score for the confidence interval of 95%

d = allowable error of 5%

Power = 90% Cases: Control = 1: 2

Based on prior research by Aziz et al. [11], using referral status as the variable of interest, 95 cases and 190 controls would be enough for statistically significant analysis.

### Sampling

Initially, all 13 health facilities with CFR of more than 1% in the region were sampled. Random samples of 10 cases per facility were then generated using random number generator. For each case, two controls admitted on same day as case were selected using same sampling approach.

### Data abstraction

In the sampled facilities, medical records of 101 cases and their controls were sampled for data abstraction. Clinicians and health information officers, under the supervision of a clinical epidemiologist abstracted information including patient's age, sex, clinical information (Table 1), date of admission and discharge, outcome, referral status, treatment given, and the physician who saw the client most frequently.

### Data analysis

Descriptive data were analysed using frequencies, proportions, medians and means. The exposure variables were age, sex, duration of stay, referral status, signs and symptoms, treatment given, and cadre of caregivers. Logistic regression at p-value < 0.05 significance level was used to assess the association between malaria mortality and its predictors. Odds ratios with 95% confidence intervals were computed to determine variables associated with malaria mortality. Confounders were accounted for by using multivariable logistic regression. Only variables that were significant in the bivariate analysis were included in the multivariate analysis.

**Table 1:** Definition of variables

Variables	Definition
Duration of stay	How long did the patient stay in the health facility before the outcome?
Referral status	Whether a different health facility sent or transferred the patient, or not, to the study facility
Presenting symptoms	Clinical complaints by the patients or their caregivers documented by the health worker
Presenting signs	The health workers documented clinical observations of the patient
Symptoms	
Fever	A complaint of a rise in body temperature by the patient or caregiver
Headache	A complaint of pain or discomfort in the face or head by the patient or caregiver
Abdominal pain	A complaint of discomfort or abnormal sensation that is felt around the belly region (abdomen) by the patient or caregiver
Loss of appetite	A complaint of lack or reduced desire to eat by the patient or caregiver
Chills	A complaint of feeling of coldness accompanied with or without fever by the patient or caregiver
Generalised body and joint pain	A complaint of pain or discomfort in the body or joints by the patient or caregiver
Bitterness in the mouth	A complaint of an unpleasant taste in the mouth by the patient or caregiver
Signs	
Severe pallor	Extreme paleness of conjunctiva or palm/sole as determined by the prescriber
Febrile	The observed rise in temperature confirmed via the use of a thermometer
Deep and fast breathing	Observed abnormal breathing, which is faster than normal per the age bracket
Rapid pulse	Observed higher heart rate per age bracket

## Results

The median age of cases and controls was 4.1 years (IQR = 21.6) and 5.7 years (IQR = 18.2) respectively, with children aged less than 5 years forming the majority of cases 71 (74.7%) and controls 100 (52.6%). Physician Assistants saw a majority 207 (72.6%) of the participants, managing 64 (67.4%) of cases and 143 (75.3%) of controls (Table 2). Concerning signs and symptoms, fever was the most prevalent 226 (79.3%) symptom among participants; 69 (72.6%) of cases and 157 (82.6%) controls presented with fever. Nearly forty percent (113) of participants did not receive malaria testing, with an almost equal proportion among cases 36 (37.9%) and controls 77 (40.5%). Only 3 (1.1%) participants were referred, and this occurred only among the cases. About a third 35 (36.8%) of cases presented with severe pallor, but this was much lower 31 (16.0%) among controls (Table 3).

**Table 2:** Socio-demographics of cases and controls, Northern Region, Ghana

Variable	Cases n (%) N = 95	Controls n (%) N = 190	Total n (%) N = 285
Age(years)			
Median age (years)	4.1 (IQR = 21.6)	5.7 (IQR = 18.2)	
Less than 5	71 (74.7)	100 (52.6)	171 (60.0)
5 and above	24 (25.3)	90 (47.4)	114 (40.0)
Sex			
Female	54 (56.8)	93(49.0)	147 (51.6)
Male	41(43.2)	97(51.0)	138 (48.4)
Prescriber type			
Medical officer	20 (21.1)	27 (14.2)	47 (16.5)
Nurse prescriber	11 (11.6)	20 (10.5)	31 (10.9)
Physician assistant	64 (67.4)	143 (75.3)	207 (72.6)

**Table 3:** Signs, symptoms, diagnosis, and treatment factors of cases and controls, Northern Region, Ghana

Variable	Cases n (%) N = 95	Controls n (%) N = 190	Total n (%) N = 285
Duration of stay			
< 24 h	50 (52.6)	22 (11.6)	72 (25.3)
≥ 24 h	45 (47.4)	168 (88.4)	213 (74.7)
Referral status			
Referred	3 (3.2)	0 (0.0)	3 (1.1)
Not referred	92 (96.8)	190 (100.0)	282 (98.9)
Presenting signs			
Severe pallor	35 (36.8)	31 (16.0)	66 (23.2)
Febrile	24 (25.3)	55 (28.9)	79 (27.7)
Deep and fast breathing	1 (1.1)	0 (0.0)	1 (0.4)
Rapid pulse	1 (1.1)	0 (0.0)	1 (0.4)
Presenting symptoms			
Fever	69 (72.6)	157 (82.6)	226 (79.3)
Nausea/vomiting	39 (41.1)	77 (40.5)	116 (40.7)
Abdominal pain	23 (24.2)	51 (26.8)	74 (26.0)
Loss of appetite	16 (16.8)	25 (13.2)	41 (14.4)
Headache	7 (7.4)	31 (16.3)	38 (13.3)
Chills	8 (8.4)	10 (5.3)	18 (6.3)
Generalized body and joint pains	6 (6.3)	9 (4.7)	15 (5.3)
Bitterness in the mouth	0 (0.0)	4 (2.1)	4 (1.4)
Test-status			
Not tested	36 (37.9)	77 (40.5)	113 (39.6)
Tested	59 (62.1)	113 (59.5)	172 (60.4)
Test result			
Test-negative	11 (18.6)	22 (19.5)	33 (19.2)
Test-positive	48 (81.4)	91 (80.5)	139 (80.8)
Additional diagnosis			
Sepsis	18 (19.0)	16 (8.4)	34 (11.9)
Gastroenteritis	10 (10.5)	38 (20.0)	48 (16.8)
Respiratory tract infection	14 (14.7)	31 (16.3)	45 (15.8)
Treatment received			
IM/IV artesunate/artemether	7 (7.4)	32 (16.9)	39 (13.7)
Quinine	57 (60.0)	93 (48.9)	150 (52.6)
Artemether/Lumefantrine or Amodiaquine	29 (30.5)	94 (49.5)	123 (43.2)
Antibiotics	43 (42.3)	84 (44.2)	127 (44.6)
Blood transfusion	18 (19.0)	21 (11.1)	39 (13.7)

At a crude level, the patient's age [odds ratio 2.66, 95% CI (1.55–4.58)] and the duration of their stay in the health facility [odds ratio 8.48, 95% CI (4.66–15.46)] were significantly associated factors (Table 4). Presenting with symptoms such as headache [odds ratio 0.41 95% CI (0.17–0.96)], signs like severe pallor [odds ratio 3.09 95% CI (1.75–5.47)], gastroenteritis [odds ratio 0.47 95% CI (0.22–0.99)], and sepsis as an additional diagnosis [odds ratio 2.54 95% CI (1.23–5.25)]. (Table 5), receiving medicines such as intravenous/intramuscular [odds ratio 0.5, 95% CI (0.3–0.8)], and receiving artemether-lumefantrine or artesunate-amodiaquine [odds ratio 0.5, 95% CI (0.3–0.8)] (Table 6) were the factors significantly associated with malaria mortality.

**Table 4:** Association between socio-demographics, referral status, duration of stay, and malaria mortality, Northern Region, Ghana

Variables	Cases n (%)	Controls n (%)	Odds ratio 95% CI	P-value
Age(years)				
Less than 5	71 (74.7)	100 (52.6)	2.66 (1.55–4.58)	0.000*
5 and more	24 (25.3)	90 (47.4)		
Sex				
Female	54 (56.8)	93 (49.0)	1.37 (0.84–2.26)	0.258
Male	41 (43.2)	97 (51.0)		
Prescriber type				
Medical officer	20 (21.1)	27 (14.2)	1.00	0.300
Nurse prescriber	11 (11.6)	20 (10.5)	0.74 (0.29–1.89)	
Physician assistant	64 (67.4)	143 (75.3)	0.60 (0.32–1.16)	
Duration of stay				
< 24 h	50 (52.6)	22 (11.6)	8.48 (4.66–15.46)	0.000*
≥ 24 h	45 (47.4)	168 (88.4)		
Referral status				
Referred	3 (3.2)	0 (0.0)		0.109
Not referred	92 (96.8)	190 (100.0)

**Table 5:** Association between patient symptoms, diagnosis, and malaria mortality, Northern Region, Ghana

Variables	Cases n (%)	Controls n (%)	Odds ratio 95% CI	P-value
Signs				
Severe pallor				
Yes	35 (36.8)	31 (16.0)	3.09 (1.75–5.47)	0.000*
No	60 (63.2)	159 (84.0)		
Deep and fast breathing				
Yes	1 (1.1)	0 (0.0)		0.335
No	94 (98.9)	190 (100.0)		
Rapid pulse				
Yes	1 (1.1)	0 (0.0)		0.333
No	94 (98.9)	190 (100.0)		
Febrile				
Yes	24 (25.3)	55 (28.9)	0.83 (0.47–1.45)	0.575
No	71 (74.7)	135 (71.1)		
Symptoms				
Fever				
Yes	69 (72.6)	157 (82.6)	0.56 (0.31–1.00)	0.062
No	26 (27.4)	33 (17.4)		
Bitterness in the mouth				
Yes	0 (0.0)	4 (2.1)		0.305
No	95 (100.0)	186 (97.9)		
Nausea/vomiting				
Yes	39 (41.1)	77 (40.5)	1.02 (0.62–1.69)	1.000
No	56 (58.9)	113 (59.5)		
Generalized body and joint pains				
Yes	6 (6.3)	9 (4.7)	1.36 (0.47–3.93)	0.580
No	89 (93.7)	181 (95.3)		
Abdominal pain				
Yes	23 (24.2)	51 (26.8)	0.87 (0.49–1.54)	0.670
No	72 (75.8)	139 (73.2)		
Loss of appetite				
Yes	16 (16.8)	25 (13.2)	1.34 (0.68–2.64)	0.474
No	79 (83.2)	165 (86.8)		
Headache				
Yes	7 (7.4)	31 (16.3)	0.41 (0.17–0.96)	0.042*
No	88 (92.6)	159 (83.7)		
Chills				
Yes	8 (8.4)	10 (5.3)	1.66 (0.63–4.34)	0.311
No	87 (91.6)	180 (94.7)		
Co-morbidities				
Gastroenteritis				
Yes	10 (10.5)	38 (20.0)	0.47 (0.22–0.99)	0.045*
No	85 (89.5)	152 (80.0)		
Respiratory tract infection				
Yes	14 (14.7)	31 (16.3)	0.89 (0.45–1.76)	0.863
No	81 (85.3)	159 (83.7)		
Sepsis				
Yes	18 (19.0)	16 (8.4)	2.54 (1.23–5.25)	0.012*
No	77 (81.0)	174 (91.6)

**Table 6:** Association between clinical/treatment factors and malaria mortality, Northern Region, Ghana

Variables	Cases n (%)	Controls n (%)	Odds ratio 95% CI	P-value
Test-status				
Not tested	36 (37.9)	77 (40.5)	0.90 (0.54–1.48)	0.700
Tested	59 (62.1)	113 (59.5)		
Test result				
Test-negative	11 (18.6)	22 (19.5)	0.95 (0.42–2.12)	1.000
Test-positive	48 (81.4)	91 (80.5)		
Treatment				
IM/IV artemether/artesunate				
Yes	7 (7.4)	32 (16.9)	0.39 (0.17–0.92)	0.029*
No	88 (92.4)	157 (83.1)		
Quinine				
Yes	57 (60.0)	93 (48.9)	1.56 (0.95–2.58)	0.080
No	38 (40.0)	97 (51.1)		
A/L OR A/Amod				
Yes	29 (30.5)	94 (49.5)	0.45 (0.27–0.76)	0.002*
No	66 (69.5)	96 (50.5)		
Antibiotics				
Yes	43 (42.3)	84 (44.2)	1.04 (0.64–1.71)	0.900
No	52 (54.7)	106 (55.8)		
Blood transfusion				
Yes	18 (19.0)	21 (11.1)	1.88 (0.95–3.73)	0.099
No	77 (81.0)	169 (88.9)		

After adjustment, age [aOR 2.8 (1.4–5.6)], duration of stay [aOR 12.0, 95% CI (5.9–24.6)], severe pallor [aOR 2.3, 95% CI (1.1–4.6)], administration of oral artemether-lumefantrine or artesunate-amodiaquine [aOR 0.4, 95% CI (0.2–0.9)], and sepsis as an additional diagnosis [aOR 4.1, 95% CI (1.8–9.5)] remained significantly associated with malaria mortality (Table 6). The odds of malaria death were higher in under-fives compared to persons aged 5 or more years (p < 0.01).

Patients who spent less than 24 h had significantly higher odds of dying than those who spent more than 24 h (p < 0.01). Patients with severe pallor had four times higher odds of dying than those without (p 0.02). Having sepsis as an additional diagnosis was associated with higher malaria mortality (p < 0.01). Table 7 also shows that participants who received oral artemether-lumefantrine or artesunate-amodiaquine had reduced odds of dying compared to those who did not receive these medicines (p = 0.02).

**Table 7:** Factors associated with malaria mortality in the Northern Region

Variable	Adjusted Odds Ratio	p-value
Age (years)		
Less than 5	2.8 (1.4–5.6)	0.003
5 or more		
Duration of stay		
< 24 h	12.0 (5.9–24.6)	< 0.0001
≥ 24 h		
IV/IM artesunate and artemether		
Yes	1.0 (0.3–2.9)	0.99
No		
Art/lum OR Art/Amod		
Yes	0.4 (0.2–0.9)	0.02
No		
Severe pallor		
Yes	2.3 (1.1–4.6)	0.02
No		
Sepsis		
Yes	4.1 (1.8–9.5)	0.001
No		
Headache		
Yes	0.7 (0.2–1.9)	0.54
No		

## Discussion

The purpose of this study was to determine the factors associated with mortality among patients with severe malaria in the Northern region of Ghana, which had a high malaria case fatality rate. Knowledge of the factors was necessary to plan malaria mortality reduction interventions. The study found significant associations between malaria mortality and factors such as the patient's age, duration of stay in the health facility, presence of severe pallor, receipt of sepsis as an additional diagnosis, and administration of oral artemether lumefantrine or artesunate amodiaquine. A prospective cohort study in the same region (Northern region) which examined the patient’s socio-demographic characteristics and clinical care factors associated with malaria mortality, found that factors such as income, educational level, occupation and marital status strongly correlated with bed net ownership and usage as a drive to reduce malaria mortality [11].

Children aged below 5 years made up the majority of study participants [12], and this finding may be due to the low immunity of this age group. The observed younger age in this study is contrary to the current trend of data reporting increasing age with malaria epidemiological transition [13], which is explainable since this data was collected about a decade ago. Due to the documented vulnerability of younger children to severe malaria and malaria mortality, under-fives are primary targets of many key interventions, such as seasonal malaria chemoprevention (SMC) and intermittent preventive treatment for malaria among infants.

The results of this study, similar to previous studies [14, 15], also showed that women made up the majority of patients admitted with severe malaria, despite the lack of a significant relationship between sex and malaria mortality. These increased numbers of cases of severe malaria in women may be due to lower immunity to malaria and a lower haemoglobin level among women. According to Quaresima et al*.* [9], women are more susceptible to malaria infections than men, primarily because of their prolonged exposure to mosquito bites during the night, when mosquitoes are most active, particularly in rural Ghana, where females often perform household chores at night.

A physician assistant managed most of the study participants, and the cadre of health workers caring for the patients had no influence on the outcome. In another study in Ghana, physician assistants also saw the majority of the malaria cases [16]. The doctor-patient ratio in a predominantly rural region of Ghana is about 1:18,257, compared to 1:4,099 in an urban region. These rural–urban inequities significantly account for some of the health outcomes across the country [6]. As a result, the majority of rural facilities have more physician assistants and nurse prescribers attending to malaria cases than medical doctors. To ensure quality of care by all cadres at all levels, the National Malaria Elimination Programme/Ghana Health Service regularly distributes to its health facilities updated malaria case management guidelines and trains not only medical officers but all cadres of caregivers. This practice may have influenced physician assistants and nurses’ ability to manage severe malaria cases.

The majority of cases spent less than 24 h in the health facility, and the duration of stay was significantly associated with malaria mortality. More than half of the cases died within 24 h of presenting at the health facility, similar to results found by other researchers [6]. The first 24 h of severe illness are critical and significantly related to the malaria admission outcome. This could be due to a patient's delay in seeking care, delay in referral to a higher level, or the prescriber's suboptimal management of cases. This study could not tell exactly why the relationship existed, but a number of studies have shown that the longer a patient with severe malaria stays away without proper medical consultation by a professional healthcare provider, the greater the patient's risk of mortality [17, 18]. The delay in the consultation may be due to the hospital's physical accessibility and the region's health insurance coverage. These issues are quite prevalent in the study region and may have contributed to the findings.

Delayed diagnosis and treatment by prescribers are also significantly associated with malaria mortalities in some studies [17, 18]. Malaria is an emergency because of its capability to progress to severe disease and then mortality if not treated appropriately and promptly [19]. Prompt diagnosis and treatment are, therefore, crucial to prevent the progression of the disease to a severe form and ultimately lower mortality. Some prescribers had documented suboptimal management and could have contributed to this finding.

Research has linked malaria mortality to delays in referral to a higher-level facility [20]. In this study, only three participants received referrals. At least a district hospital or polyclinic should manage severe malaria cases, according to the national malaria case management guidelines. The low referral rate raises concerns because it could indicate that lower-level health facilities are managing cases beyond their capacity, potentially resulting in adverse patient outcomes. The long inter-facility distance and poor state of roads in the study region may also contribute to the low referral rate for severe malaria cases in this study.

The most recorded sign was fever, followed by severe pallor. The presence of severe pallor significantly increased the odds of malaria mortality. Previous studies [21, 22] found similar results. Interestingly, the number of blood transfusions done was low compared with the number of severe pallors. Researchers have found that blood transfusions reduce malaria mortality, particularly in the under-five age group [23]. The observed difference in severe pallor and blood transfusion could have resulted from the lack of confirmation of the perceived anaemia (severe pallor) by haematological analyses. The attending health workers may have misread the level of pallor, since that is subjective. The possibility of case mismanagement, which could result in mortality, cannot be ignored.

The most frequently associated diagnoses and presentations were gastroenteritis, respiratory tract infections, and sepsis. Most of the time, severe malaria cases in this study had non-specific symptoms and diagnoses that were similar to those seen in SSA [24–26]. Like other studies [27], having comorbidities (sepsis) was independently associated with severe malaria and mortality. This could complicate the malaria case and, if not managed well, lead to death. Contrary to another study, abnormal breathing did not emerge as an important marker of mortality [27].

Interestingly, administration of parenteral artesunate or amodiaquine, the preferred drugs for treating severe malaria, was not associated with reduced odds of malaria death. However, administration of oral artesunate amodiaquine and artemether lumefantrine seemed to have rather lowered the odds of death in persons with severe malaria. According to World Health Organization treatment guidelines and other studies, a combination of parenteral malaria treatment and an artemisinin-based combination significantly reduced malaria mortality [2, 28]. This study did not account for the severity of the infection potentially resulting in the administration of parenteral treatment to cases with a high risk of death, while milder cases likely received oral anti-malarial treatment. Other system factors, such as stockouts, may have influenced the treatment modality chosen.

### Limitations of the study

Without a laboratory test, about 38% of cases and 41% of controls received a clinical diagnosis. This raises the possibility of misclassification because malaria symptoms mimic those of many other febrile illnesses. Further, the use of secondary data limited the study to the variables reported in patients' records. Although the study could not explore the effect of additional variables on the outcome as well as their effect on the associations, the findings presented in this paper provide important clues on the socio-demographic and clinical care drivers of malaria mortality in the northern region of Ghana. Hence this paper provides evidence that could influence policy decisions regarding the management of severe malaria across geographies where they may apply.

## Conclusion

This study sought to determine factors associated with mortality among patients with severe malaria in the Northern region of Ghana. Malaria mortality was associated with being less than 5 years of age, severe pallor, sepsis as an additional diagnosis, a short stay in the health facility, and the use of oral anti-malarial medicines. The National Malaria Elimination Programme and its partners should implement measures to strengthen the referral system, including the provision of at least one ambulance for each health centre and district hospital and the sensitization of health workers on referrals. There should be a review of malaria control interventions and management targeted at children under five in the region. Health workers should undergo retraining in managing malaria cases. Future studies should explore factors accounting for the late reporting for health services and the delay or non-referral to higher-level facilities.

## Data Availability

Data on which study conclusion were drawn have been made available in this paper and attached supplement. The datasets used and/or analysed are available from the corresponding author on reasonable request.

## Abbreviations

NMEP: National Malaria Elimination Programme
CFR: Case fatality rates
CHPS: Community Health and Planning Services
PPMED: Directorate of Policy, Planning, Monitoring and Evaluation Division
AL: Artemether lumefantrine
AA: Artesunate amodiaquine
SMC: Seasonal malaria chemoprevention
GHS: Ghana Health Service
